# Bodyweight and body condition scores of Australian British shorthaired cats, 2008–2017

**DOI:** 10.3389/fvets.2023.1241080

**Published:** 2023-10-11

**Authors:** B. J. Murphy, M. A. Stevenson, C. S. Mansfield

**Affiliations:** Melbourne Veterinary School, Faculty of Science, The University of Melbourne, Werribee, VIC, Australia

**Keywords:** British shorthair, cats, obesity, body weight, body condition, prevalence, survival

## Abstract

Obesity is the most common nutritional problem in companion animals today, and Australian British shorthair (BSH) cats have been shown to have a greater likelihood of being overweight relative to other cat breeds. The objectives of this research were to quantify bodyweight (BW) and body condition scores (BCSs) of BSH cats attending first opinion practice in Australia for the period 2008–2017 and to determine if: (1) being classified as overweight was associated with geographical location (urban versus rural and socio-economic index); and (2) BW recorded in the first 12 months of life was associated with length of life beyond 12 months. Electronic medical records from BSH cats were obtained from VetCompass Australia and used for BW and BCS analysis. Desexed males ( n = 971) had the highest mean BW followed by entire males ( n = 79), desexed females ( n = 815), and entire females ( n = 82). The desexed males, desexed females, and entire females had a mean BCS classified as overweight using a 1-to-9 point BCS scale. The entire male population was the only group with a mean BCS classified as ideal. No statistically significant association between BW and urban-rural status and no consistent trend between BW and socioeconomic deprivation was found. For cats with at least one BW measurement in the first 12 months of life that was ≤3.3 kg, the age when 20 percent of the group had died or were euthanised was 12.3 (95% CI 11.7 to 13.1) years. For cats with at least one BW measurement in the first 12 months of life that was ≥3.3 kg age, the age when 20 percent of the group had died or were euthanised was 6.6 (95% CI 5.2 to 6.6) years. This was a substantial clinical difference in survival. The study concluded that a large proportion of BSH cats attending first opinion veterinary clinics in Australia between 2008 and 2017 (48%) were classified as overweight. Cats less than 12 months of age that were greater than 3.3 kg had a shortened lifespan beyond 12 months of age compared with cats that were less than 3.3 kg.

## 1. Introduction

Obesity is one of the most common medical disorders of pet cats (1, 2) and is regarded as a significant welfare issue (3), given its associations with multiple comorbidities (2, 4) and mortality (5). Cats in underweight condition also have a greater overall mortality risk and higher morbidity (5, 6). As a result, regular assessment of body weight and body condition score (BCS) are important for establishing a cat’s health status, as well as for ensuring accurate medication dosing. The early identification of changes in either body weight or BCS, or both, enable prompt action, in terms of both diagnostic investigations and intervention.

Australian British shorthair (BSH) cats have been shown to have a greater likelihood of being overweight relative to mixed breed cats (6) and to have higher body condition scores (BCS) relative to other purebred cats (1). A cross-sectional study by Gates et al. in New Zealand using 47,521 medical records collected between 1 January 2011 and 30 June 2016 from 10 primary care clinics found 36% of BSH cats were either overweight or obese (5). With obesity well-recognised as a risk factor for insulin resistance and diabetes mellitus (7) it is no surprise that a study conducted by O’Neill et al. (7), found that BSH cats in England were one of the most common breeds diagnosed with diabetes mellitus. At the other end of the body condition score (BCS) spectrum, weight loss and sarcopenia has been associated with increased prevalence of hyperthyroidism, chronic kidney disease, inflammatory bowel disease, neoplasia and diabetes mellitus among older cats (8, 9).

Clinically, BW is a precise, repeatable, and objective measurement (10). Tracking BW is a sensitive tool for monitoring health status in Australian BSH cats allowing trends to be identified and acted on. Ideal BWs for the adult cat ranges between 2 and 7 kg (4, 10). The reasons for this variation in ideal adult BW is that BW differs between breeds and sexes and is subject to individual variation (10). The use of BCS alongside BW helps to assess the cat’s current overall condition (emaciated, underweight, ideal, overweight, obese) (9). Considering the predisposition of BSH cats to obesity and diabetes mellitus, recording BW and BCS are particularly important for monitoring and maintaining good health in this breed (1, 6, 7). Muscle condition score (MCS) monitoring at the veterinary visit is also an important tool for identifying and preventing sarcopenia in senior cats or cats with chronic diseases resulting in loss of muscle mass, however, this was not able to be assessed in this study (10).

The aim of this study was to quantify BW and BCS measurements among Australian BSH cats using VetCompass data collected from cats presenting to first-opinion veterinary practices. Recorded BCS were interpreted according to the World Small Animal Veterinary Association (WSAVA) Global Nutrition Committee (GNC) guidelines (11). These data were then used to establish the association between BW and longevity and to determine if there were differences in BWs among cats living in urban versus rural areas and cats living in areas of high and low socio-economic status.

## 2. Materials and methods

### 2.1. Study design and data collection

This was a retrospective cohort study using consultation records for BSH cats presented for treatment and routine health care procedures at Australian primary care veterinary clinics. The data were collated by VetCompass Australia (12) for the period 2 January 2008 to 31 December 2017 and included a total of 113,596 consultation records. For each consultation record the following data were retrieved: the anonymised unique patient identifier, consultation number, consultation date, breed, sex, coat colour, date of birth, reproductive status, the date on which the cat died or was euthanased (for cats that were deceased), the postcode of the owner’s place of residence, BW, BCS, and clinical examination details (including body temperature, heart rate, respiratory rate, dental grade, pain score, capillary refill time, mucous membrane colour and blood pressure, where appropriate).

Ethics approval for this study was provided by the University of Sydney Research Integrity and Ethics Administration (Project number: 2013/919).

### 2.2. Data processing

**Figure 1:**
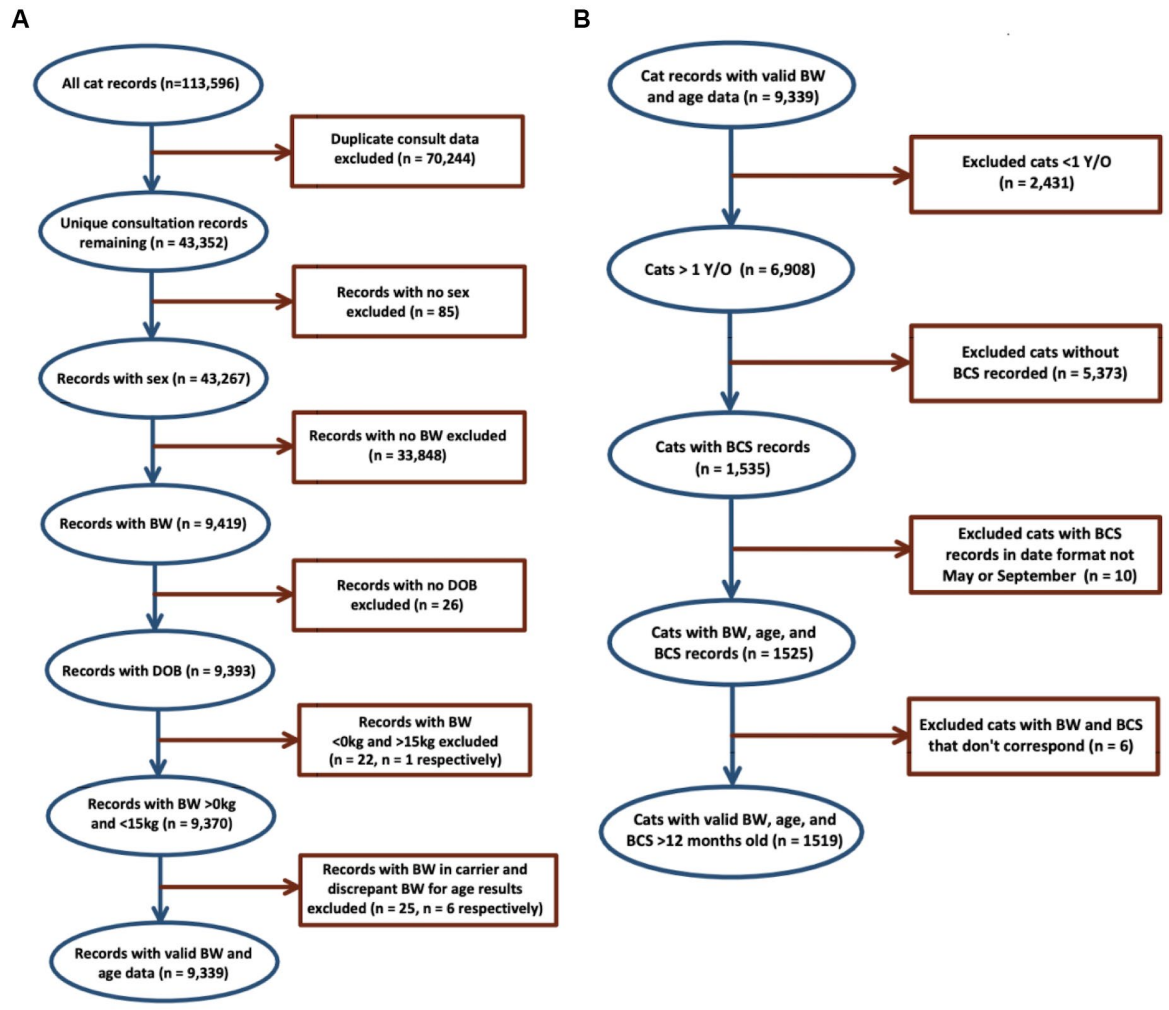
Bodyweight and body condition scores of Australian British shorthair cats, 2008–2017. Flow diagram of inclusion and exclusion criteria: **(A)** details of data cleaning of 113,596 records produced the dataset of 9,339 BW records from 2,540 cats for evaluation of BW of the population; **(B)** dataset of 1,519 records from 810 cats produced for evaluation of BCS of the population.

Data cleaning was carried out using Microsoft Excel 2021 (13). Duplicate consultation entries were excluded, followed by records without patient sex, date of birth and at least one BW measurement. Records with BW >15 kg were excluded on the basis that these were outside of the biologically plausible range, had discrepant BCS to BW data, and were likely due to errors in data entry (8). Following data cleaning, a dataset of 9,339 consultation records from 2,540 cats was produced. Figure 1 provides a detailed description of exclusions that led to the final data set.

Using the cleaned data, records of cats <12 months old, lacking BCS, data entry errors, and with discrepant BCS to BW data were excluded, leaving a total of 1,519 consultation records for BCS analysis. BCS records scored on a five-point scale were converted to a five-point scale by multiplying by 1.8.

### 2.3. Statistical analyses

Age at the time of each BW measurement was calculated as the difference (in days) between date of birth and the date of consultation. Using BCS records cats were classified as emaciated (BCS 1), underweight (BCS 2 to 3), ideal (BCS 4 to 5), overweight (BCS 6 to 7) or obese (BCS 8 to 9) (11, 14). Scatterplots were developed to show the relationship between age and bodyweight by sex and reproductive status. The hypothesis that there was no difference in BWs for cats according to sex and reproductive status was tested using the student’s t -test.

Using the postcode of residence recorded for their owner ( n = 2,272) cats with both BW and postcode data were assigned an “urban” or “rural” classifier using the Australian postcode classification search tool provided by the Commonwealth Department of Agriculture, Water and the Environment (15). Postcodes of residence with an Index of Relative Socio-economic Disadvantage (IRSED) available ( n = 8,542 records; n = 2,263 cats) were used to group records by IRSED decile (with 1 representing low socio-economic areas and 10 representing high socio-economic areas) (16). The hypothesis that there was no difference in BWs for cats from urban and rural areas was tested using the student’s t -test. The hypothesis that there was no difference in BWs for cats from each of the 10 IRSED decile areas was tested using a one-way analysis of variance. Rural and urban groups and IRSED decile areas were further separated into age, sex and neuter status to test for association with BW, but no substantial differences were identified. We therefore elected not to report these findings in the results section.

Survival analyses were used to quantify the association between the maximum BW recorded during the first 12 months of life and survival beyond 12 months of age. Identifying overweight animals when <12 months of age to assess survival was done so on the basis that age related disease and illness would have a confounding effect on survival times. Consultation records for all cats where at least one BW measurement was recorded at less than 12 months of age were selected. These records were then grouped by cat to return the maximum BW recorded for each individual during their first year of life. Descriptive statistics were computed for these BWs and the 75th quantile (3.3 kg) selected as the cut point to classify cats into “normal” and “overweight” groups. Kaplan–Meier survival curves were developed to describe time to death post 12 months for the normal and overweight cat groups. Here, the outcome of interest was the age at which the cat died or was euthanised (as recorded in their consultation records). Cats that had not died or had no record of death in their consultation records were censored on the date of their last recorded consultation.

Microsoft Excel version 16.5013 was used for data cleaning and descriptive statistics. Jamovi version 1.6.23.017 using the contributed scatterplot (17) and deathwatch (17) packages was used for the survival analyses.

## 3. Results

### 3.1. Bodyweight and body condition score

The dataset comprised BW records from more males (55%; n = 3,837) than females (45%; n = 3,071) and more desexed (92%; n = 6,585) than entire (8%; n = 323) cats (Table 1). Cats <12 months contributed the largest number of BW records, at 26%, likely correlating to kitten vaccinations and neutering. The number of records more than halved to 12% in the 1 year to 2 years old age groups.

**Table 1:** Bodyweight and body condition scores of Australian British shorthair cats, 2008–2017.

Strata	n cats	n records	Mean (SD)	*Q*1, *Q*3	Min, max
**Age**					
Juvenile	1,094	2,431	2.6 (1.2)	1.6, 3.3	0.1, 9.6
Adult	1,947	6,908	5.4 (1.3)	4.5, 6.1	1.0, 14.3
Total	2,540	9,339	4.6 (1.8)	3.6, 5.8	0.1, 14.3
**Sex (adults)**					
Male	1,351	3,837	5.9 (1.3)	5.0, 6.6	1.4, 14.3
Female	1,189	3,071	4.8 (1.1)	4.1, 5.5	1.0, 13.8
Total	1,947	6,908	5.4 (1.3)	4.5, 6.1	1.0, 14.3
**Reproductive status (adults)**					
Female neutered	815	2,928	4.8 (1.1)	4.1, 5.5	1.0, 13.8
Female entire	82	143	4.6 (1.3)	3.7, 5.5	1.3, 8.2
Male neutered	971	3,657	5.8 (1.3)	5.0, 6.6	1.4, 13.1
Male entire	79	180	5.2 (1.3)	4.4, 5.9	3.4, 14.3
Total	1,947	6,908	5.4 (1.3)	4.5, 6.1	1.0, 14.3

Descriptive statistics of bodyweight (kg) measurements for the 2,540 cats included in this study by age, sex, reproductive status.

Records of cats <12 months ( n = 1,094 cats) were excluded for our descriptions of BW and BCS for the adult population. The adult dataset was comprised of 6,908 BWs from 1,947 cats. This included 897 females (46%) of which 815 were desexed (42%) and 82 were entire (4%). Adult males numbered 1,050 (54%) and included 971 desexed cats (50%) and 79 entire cats (4%).

The mean BW of the population (including cats <12 months old) was 4.6 (95% CI 4.6 to 4.7) kg, and the mean BW for adults was 5.4 (95% CI 5.3 to 5.4) kg. Desexed males had the highest mean BW of 5.8 (95% CI 5.8 to 5.9) kg followed by entire males (5.2; 95% CI 5.0 to 5.5 kg), desexed females (4.8; 95% CI 4.8 to 4.9 kg) and entire females (4.6; 95% CI 4.4 to 4.8 kg).

BWs for the population were normally distributed. The age group in which the highest mean BW of 5.8 (95% CI 5.7 to 6.0) kg occurred was at 9 years and the lowest mean BW was seen in the 20 years-old age group at 3.5 (95% CI 3.1 to 3.9) kg. Entire females reached maximum BW first at 4 years of age, followed by desexed males at 5 years, entire males at 6 years and finally, desexed females at 9 years.

**Figure 2:**
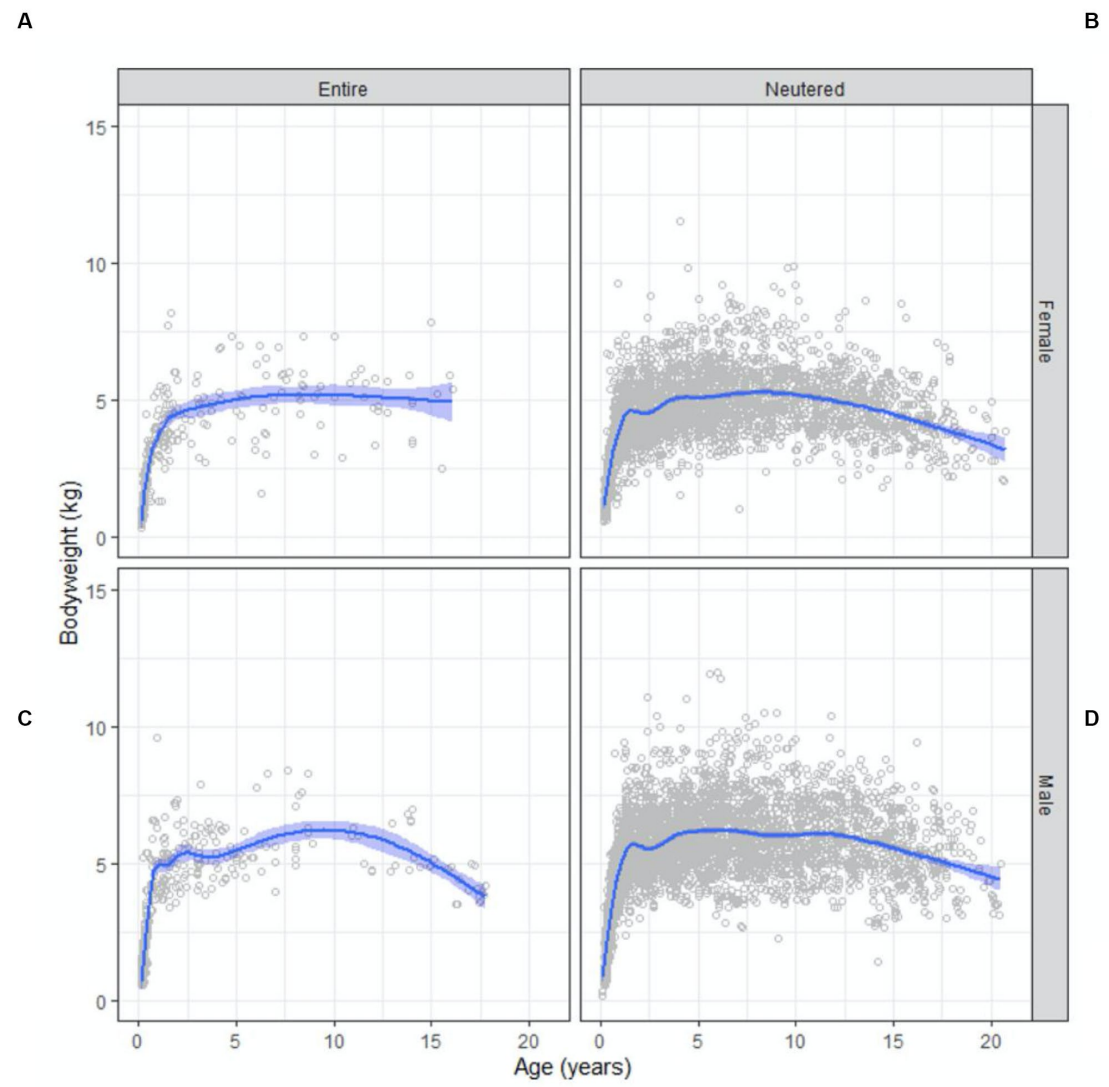
Bodyweight and body condition scores of Australian British shorthair cats, 2008–2017. Scatterplots showing bodyweight as a function of age (in years) conditioned by sex and reproductive status: **(A)** entire female population; **(B)** neutered female population; **(C)** entire male population; **(D)** neutered male population. Superimposed on each plot is a non-parametric line of best fit to the data.

Scatter plots of BW as a function of age, conditioned by sex and reproductive status are shown in Figure 2. Males were 1 kg heavier than females [5.8 (95% CI 5.7 to 5.9) and 4.8 (95% CI 4.8 to 4.9)] kg, respectively ( t -test statistic 33.1; df 6,906; *p* < 0.01). Mean BW for desexed females was 4.9 (95% CI 4.8 to 4.9) kg compared with 4.6 (95% CI 4.4 to 4.8) kg for entire females. Mean BW for desexed males was 5.8 (95% CI 5.8 to 5.9) kg compared with 5.2 (95% CI 5.0 to 5.5) kg for entire males. Differences in BW by reproductive status was statistically significant ( t -test statistic 5.7; df 6,906; *p* < 0.01).

**Figure 3:**
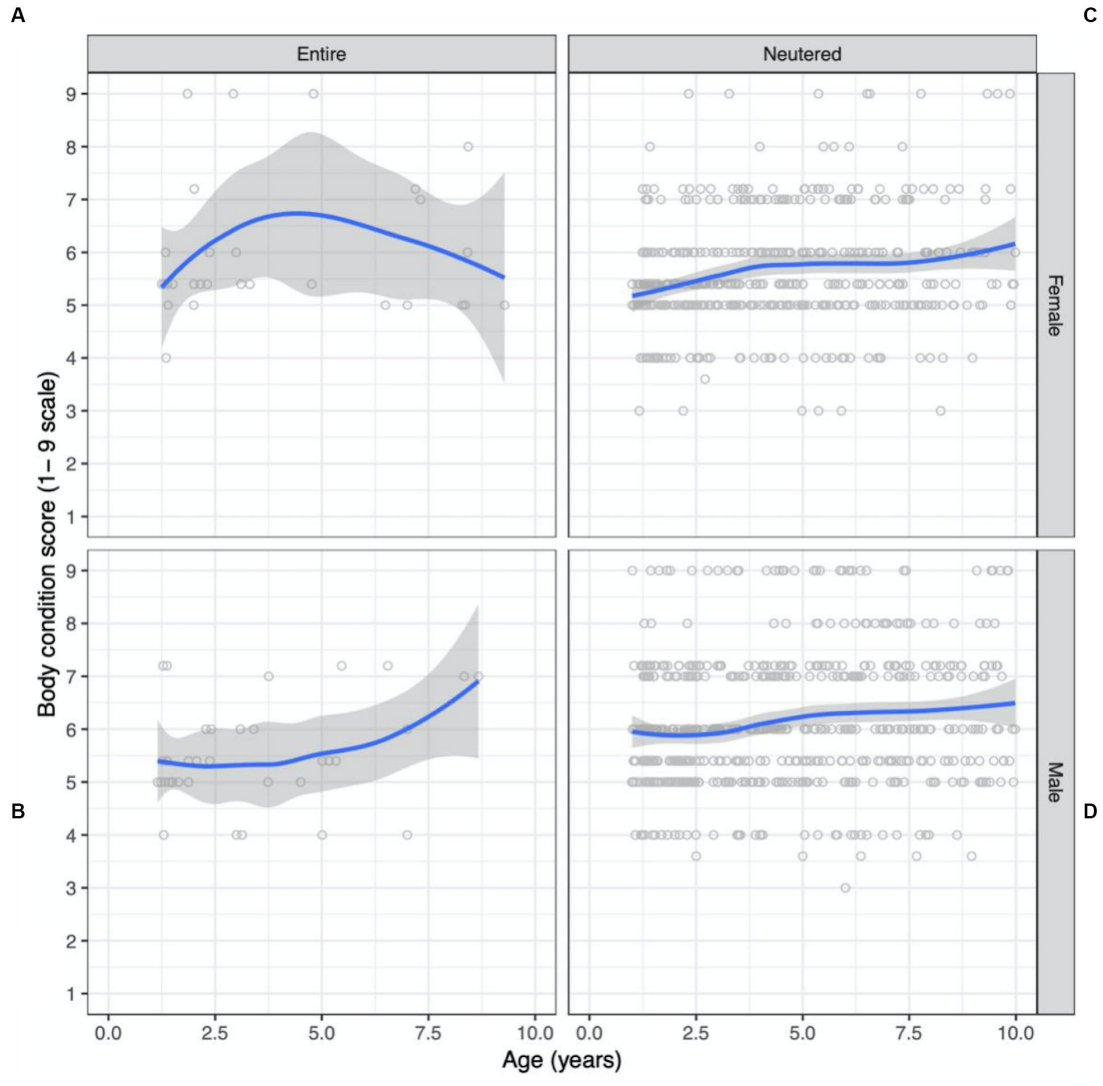
Bodyweight and body condition scores of Australian British shorthair cats, 2008–2017. Scatterplots showing body condition score (expressed on a 1–9 scale) as a function of age (in years) conditioned by sex and reproductive status: **(A)** entire female population; **(B)** neutered female population; **(C)** entire male population; **(D)** neutered male population. Superimposed on each plot is a non-parametric line of best fit to the data.

Scatterplots of BCS as a function of age, conditioned by sex and reproductive status are shown in Figure 3. The dataset consisted of BCS records from more males (56%) than females (44%) (Table 2). Desexed males contributed 54% records, desexed females contributed 41%, entire males contributed 3%, and entire females contributed 2% of BCS records. Overall, the mean BCS for the population was 5.8 (95% CI 5.7 to 5.9) on a 1-to-9 scale which approaches the overweight category (BCS 6 to 7). The prevalence of ideal BW, overweight, and obese cats for the adult population was 51%, 40%, and 7%, respectively.

**Table 2:** Bodyweight and body condition scores of Australian British shorthair cats, 2008–2017.

Strata	n cats	n records	Mean (SD)	*Q*1, *Q*3	Min, max
**Age**					
Juvenile	2,431	1,094	—	—	—
Adult	1,947	6,908	5.8 (1.3)	5, 7	1, 9
Total	2,540	9,339	5.8 (1.3)	5, 7	1, 9
**Sex (adult)**					
Female	1,189	3,071	5.5 (1.2)	5, 6	1, 9
Male	1,351	3,837	6.0 (1.3)	5, 7	1, 9
Total	1,947	6,908	5.8 (1.3)	5, 7	1, 9
**Reproductive status (adults)**					
Female neutered	815	2,928	5.5 (1.1)	5, 6	2, 9
Female entire	82	143	5.4 (2.1)	5, 6	1, 9
Male neutered	971	3,657	6.1 (1.3)	5, 7	1, 9
Male entire	79	180	5.3 (1.1)	5, 6	3, 7
Total	1,947	6,908	5.8 (1.3)	5, 7	1, 9

Descriptive statistics of body condition score estimate for the 2,540 cats included in this study by age, sex and reproductive status.

The desexed male population had the highest mean BCS, at 6.1 (95% CI 6.0 to 6.2) followed by entire females (5.7; 95% CI 5.4 to 6.0), desexed females (5.6; 95% CI 5.5 to 5.7) and entire males (5.4; 95% CI 5.2 to 5.6). All but the entire male population were classified as overweight. The desexed male population was the only group with a higher prevalence of overweight BCS than ideal BCS, with 56% overweight or obese and 43% in ideal body condition. The entire female population had the highest prevalence of obesity, at 11%, followed closely by the desexed male population at 10.6%.

### 3.2. Body weight and urban-rural location

Analyses of BWs by urban-rural classification of the postcode in which the cat lived was carried out using 8,570 BW records from 2,272 cats. Urban cats formed the majority, contributing 1,836 cats (81%) and 7,049 records (82%). The rural cohort was comprised of 436 cats (19%) and 1,521 records (18%). There was no statistically significant difference between BW and urban-rural location ( t -test statistic 0.41; df 8,569; *p* = 0.68).

### 3.3. Body weight and IRSED

There were 8,542 records from 2,263 cats used in the analysis of BW by IRSED decile. The largest contribution of records came from the most affluent group, IRSED decile 10, with 1,766 records (21%), followed by IRSED decile 9, with 1,621 records (19%). The smallest contribution was from the lowest IRSED decile group, with 221 records (2.6%). This showed a distinct correlation of high socio-economic status with a higher number of BW records, most likely reflecting the presence of more frequent veterinary visits within these areas. One way analysis of variance showed that the effect of IRSED decile on BW was statistically significant (*F* test statistic 9.24; df 9,1928; *p* < 0.01). While BW varied by socio economic index of the area in which the cat lived, there was no consistent trend in the association between BW and socioeconomic deprivation score.

### 3.4. Body weight and survival

Data from 1,950 cats were used for the survival analyses. Of this group, 249 cats (13%) cats had a maximum BW of greater than 3.3 kg in the first 12 months of life. Of the 1,950 cats 292 (15%) had a recorded date of death; the remaining 1,658 cats were censored observations.

**Figure 4:**
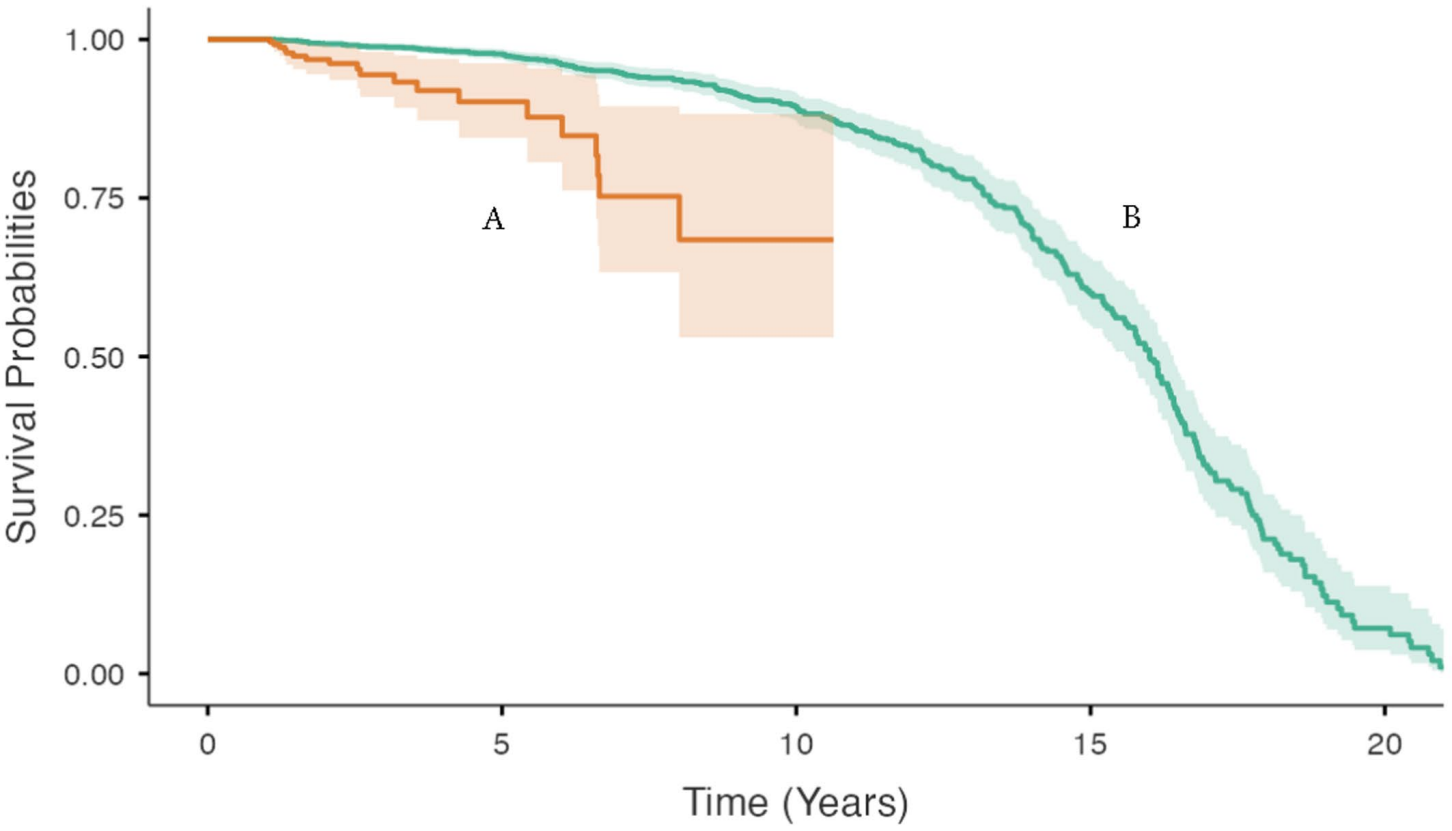
Bodyweight and body condition scores of Australian British shorthair cats, 2008–2017. Kaplan–Meier survival curve showing the cumulative proportion of cats surviving as a function of age (in years) for: **(A)** cats with a maximum BW >3.33 kg in the first 12 months of life (orange); and **(B)** cats with a maximum BW ≤3.33 kg in the first 12 months of life (green). Ninety five percent confidence intervals around each survival curve are indicated by the respective shaded areas.

For cats with at least one BW measurement in the first 12 months of life that was <3.3 kg, the age when 20 percent of the group had died or were euthanised was 12.3 (95% CI 11.7 to 13.1) years. For cats with at least one BW measurement in the first 12 months of life that was ≥3.3 kg age, the age when 20 percent of the group had died or were euthanised was 6.6 (95% CI 5.2 to 6.6) years (Figure 4).

## 4. Discussion

### 4.1. Bodyweight and body condition score

Based on the data presented in this study we conclude that 47.7% of the adult BSH cat population attending primary care veterinary clinics in Australia between 2008–2017 was overweight with a BCS of >6 using a 1-to-9 scale. The mean BCS of the population was 6 (95% CI 6.0 to 6.2) and the mean BW of the population was 5.4 (95% CI 5.3 to 5.4) kg. The correlation between BW and the nine-point BCS has been shown to be strong in cats, indicating that BW generally aligns with BCS, with slight differences attributed to variation in body configuration and individual fitness (6). Comparison of our BW and BCS findings to those of cats in ideal body condition (BCS 5) by Kienzle et al. (4) (Table 3) reinforces the conclusions from this study. Kienzle et al. (4) reported an adult female mean BW of 3.6 ± 0.83 kg, which is more than 1 kg lighter than the overweight mean BCS of 6 in the adult female population in this study. The mean BSH adult male BW in Kienzle et al. was reported at 5.1 ± 0.65 kg, just under 1 kg lighter than the overweight mean BCS of 6 in the adult male population in this study. It should be noted that the number of cats in the Kienzle et al. study was relatively small ( n = 23) and therefore the reported BW means may not be representative of the wider, target population of cats for that study.

**Table 3:** Bodyweight and body condition scores of Australian British shorthair cats, 2008–2017.

Outcome	Location	Study size	Strata	Result	Reference
Bodyweight	Germany	23	Female	3.6 ± 0.8 kg	Kienzle et al. (4)
	Germany	23	Male	5.1 ± 0.6 kg	
	Australia	6,908	Female	4.8 ± 1.1 kg	This study
	Australia	6,908	Male	5.8 ± 1.3 kg	This study
BCS	Australia	37		5.9 ± 0.95	Corbee et al. (18)
	Australia	1,519		5.8 ± 1.26	This study
Obesity prevalence	New Zealand	383		38%	Gates et al. (5)
	Sweden	26		65%	Öhlund et al. (3)
	Australia	1,519		48%	This study

Details of other studies that have assessed bodyweight and body condition score in BSH cats.

The BCS results reported in this study were similar to the small number of other studies that included BSH cats. The prevalence of overweight and obese adult BSH cats in our study comprised almost half the population at 48%. Gates et al. found a prevalence of 38% overweight and obese adult BSH cats in New Zealand (5), just under 10% less than our study population. In a study conducted by Öhlund et al. in BSH cats presenting to the University Animal Hospital, Swedish University of Agricultural Sciences, there was a much higher prevalence of overweight BSH cats, at 65%. This was significantly higher than the domestic cat population where the overweight prevalence was 47% (3), a prevalence consistent with our findings. Corbee et al. conducted a study of adult show cats reporting a mean BCS of 5.92 ± 0.95 in Australian BSH (18). In another study, Teng et al. (6) found the BSH breed to be a risk factor for overweight BCS, with BSH cats being 1.35 times more likely to be overweight than other breeds and domestic cats. This study was conducted using patient records from a feline primary practice in metropolitan Sydney acquired between 2005 and 2015 (Table 3).

Numerous studies have identified male cats as being at greater risk of being overweight compared with female cats (2, 3, 6, 19–23). In our study, male cats were at increased risk of overweight/obesity compared to female cats (OR = 1.94; 95% CI = 1.10, 3.40). Female cats in our study were also found to be at increased risk of being underweight compared to male cats (OR = 3.06; 95% CI = 0.28, 33.75). It is possible that this may be due to a greater proportion of elderly female cats in the population, as female cats have been reported to live longer than males (6). However, there were 1.4 times more male cats 15 years of age and over compared to females in our study population, so this was unlikely to be an influencing factor. There is also a risk of confirmation bias if an assessment of BCS is made after BW has been measured. Because male cats, in general, weigh more than females this may influence a veterinarian’s assessment of BCS (4, 6). Another possible explanation is that females have a higher daily energy requirement per kilogram of BW (24). Daily food intake requirements on pet food packaging do not provide recommendations based on sex (6), therefore, it is possible that male cats are being overfed, resulting in an increased risk of being overweight. Conversely, it is possible that female cats are being underfed, resulting in increased risk of being underweight. A recent study also found that dogs of overweight owners were >3 times more likely to be obese (25). Owner information was not available for this study, but this could be an area for future research.

In this study desexed cats were more likely to be overweight than entire cats. The small number of entire adult cats in our study limits our ability to conclude this definitively, however, numerous other studies have also concluded desexing as a highly significant risk factor for overweight/obesity in cats (3, 6, 20–22). Sex hormones play an important role in the regulation of metabolism and it has been shown that metabolic rate and energy requirements are reduced following desexing, with desexed cats requiring 10% less energy intake per day compared to entire cats (2, 21, 22, 24, 26). Appetite and daily food intake have been shown to increase, and physical activity has been shown to decrease following desexing. These factors combined with reduced metabolic rate results in a net daily energy excess and subsequent accumulation of adipose tissue (2, 6, 26). Considering these physiological changes that occur post-desexing, the age at which desexing occurs is also likely to influence BW in the adult cat. A recent study found that early desexing provoked more rapid weight gain to produce heavier skeletally mature cats when compared to late desexing, particularly in female kittens (27). Currently, early desexing of cats is widely promoted in Australia for its benefits of population control, decreased neoplasia risk, and curbing undesirable male behaviours. The risk of overweight/obesity should also be considered when determining age of desexing, particularly in predisposed breeds such as BSH. If early desexing is to be recommended by veterinarians, then better discussions around weight monitoring and management strategies should also be employed post-operatively to prevent excessive weight gain from occurring (27).

Peak BW occurred at 9 years of age (Figure 2), then began to decrease gradually after this point, and by 16 years of age the mean BW had decreased by over 1 kg to the lowest mean BW of 3.5 kg. Our findings are comparable to those of other studies of cat populations that identified middle-aged cats (those 7 to 11 years of age) as the most at risk of being overweight (3, 6). This is likely attributable to reduced physical activity and reduced metabolic rate (2, 20, 22). The steady decline in mean BW in our BSH population was also similar to other studies, where cats 15 years and older were at most risk of being underweight relative to middle-aged cats (6). The likely reasons for lighter body condition in older cats include sarcopenia (age-related muscle loss) and conditions causing weight loss that are more common in older cats, such as diabetes mellitus, dental disease, chronic kidney disease, neoplasia, inflammatory bowel disease and hyperthyroidism (3). Additionally, the more veterinarian visits cats had over the age of 11, the more likely they were to be in ideal body condition (6). Frequent visits means closer monitoring of BW and BCS, allowing action to be taken sooner to prevent weight loss in senior cats. It could also suggest a higher level of care provided by the owner, even if the reason for frequent veterinary visits is a chronic health condition (6). Either way, this strengthens the case for regular health checks to maintain body condition in senior cats. Addition of muscle condition score (MCS) monitoring at the veterinary visit is also an important tool for identifying and preventing sarcopenia in senior cats or cats with chronic diseases resulting in loss of muscle mass.

### 4.2. Body weight and urban-rural location

Several studies have identified low activity level as a risk factor for obesity and many studies have concluded that indoor cats are at increased risk of overweight/obesity due to less opportunity to expend energy through physical activity (2, 6, 21). One would also assume that a rural lifestyle is more conducive to outdoor access and subsequent higher activity levels, and therefore a lower prevalence of obesity. However, this was not the case in this study, with no statistically significant association found between rural or urban location and overweight/obesity. Colliard et al. (20) also found no statistically significant association between outdoor access and overweight/obesity. However, these cats were still living in an urban environment, so differences in energy expenditure and body condition were unlikely to be substantial (20). An Australian study found that rural and semi-rural cats were more likely to be overweight and obese compared to urban cats (23). It is possible that although rural cats expend more energy with an active, outdoor lifestyle, they may have access to other food sources, whereas urban or indoor lifestyles may have a more controlled daily food intake. A limitation in this study is that activity levels have been assumed based on cat location and were not directly measured. A more definitive conclusion could be drawn if activity level of cats was taken into consideration as well as geographical location.

### 4.3. Body weight and IRSED

There was a statistically significant association between BW and IRSED in this study but no consistent association between BW and socioeconomic circumstances of cats. It’s possible that this breed is owned by a higher socioeconomic demographic generally, as they are an expensive breed to purchase. A higher number of veterinary visits per cat was apparent in the highest IRSED categories. This could be due to wealthier owners being able to afford more regular visits to the vet. As mentioned previously, a larger number of clinic visits in cats aged 11 years and older was associated with ideal BCS and most likely a reflection of a high level of care provided by owners (6).

### 4.4. Body weight and survival

The median survival time of cats in this population (16 years) was greater than the 12 years reported in a study of 69 BSH cats by O’Neill et al. (28). In a Swedish study, the median lifespan was reported at >12.5 years, with 54% of the study population reaching this age or older (29).

In humans, obesity in childhood and youth has been shown to result in premature mortality in adulthood (30). In this study, BSH cats identified with a BW in the upper IQR in their first year of life had a shorter lifespan compared with their lighter counterparts. Assuming that the upper IQR for BW captured the group of cats that eventually became overweight adults, the results of this study imply a reduced lifespan in those cats likely to be overweight/obese as adults. These results are reflected in a study of nine-point BCS with lifespan in a cat population of 2,609 in Sydney, Australia, which concluded that a maximum BCS of 9 was negatively associated with survival, as was a maximum BCS <5.3. Because excessive fat accumulation increases the risk of chronic disease, rather than overweight/obesity being the direct cause of death, it’s more likely that a higher incidence of chronic illness resulting in reduced quality of life was responsible for decreased longevity in overweight and obese cats (19, 30).

### 4.5. Study limitations

There are several limitations to this study. The study was retrospective and as such, not all factors that help to explain obesity risk were recorded for all study subjects. Conclusions drawn from cats at the senior end of the age spectrum were limited by a small sample size, particularly for entire cats. Another limitation was that MCS was not considered in the evaluation of overall body condition. While evaluation of BCS tells us about the amount of fat and BW provides us with an objective measurement of weight, it does not tell us about the amount of muscle on an animal. It is possible that a cat can have an ideal BCS and be losing weight due to loss of muscle, for example with hyperthyroidism (10). Therefore, what appears to be ideal BCS may not actually be ideal in terms of health.

As mentioned previously, BCS is a subjective measurement, which is prone to confirmation bias if BW is measured before BCS assessment, particularly in male cats that weigh more than their female counterparts (4, 6). Data used in this study were obtained from a wide range of clinics. For a subjective measurement such as BCS, variation in technique and BCS judgement between veterinarians could result in misclassification bias, therefore reducing the internal validity of the study. However, the wide range of sources of data can also be considered a strength, as this reduces selection bias and therefore results are more likely to be representative of the general population of BSH cats throughout Australia.

## 5. Conclusion

To the authors’ knowledge, this is the largest study of BW and BCS conducted in the BSH breed to date. The Australian BSH population was found to be overweight by assessment of BW and BCS, which is consistent with findings of other studies of BSH body condition. Owner socio-economic status was not consistently associated with mean BW and geographic location was not associated with increased BW and BCS.

Young cats that were heavier in their first 12 months of life went on to have shorter lifespans relative to their lighter BW counterparts. The results of this study indicate the importance of monitoring body condition with BW, BCS, and MCS not only in BSH cats, but all cats. Early identification of decreased body condition can signify presence of disease or age-related sarcopenia. Identifying an increase in body condition allows early action to be taken to prevent the progression into overweight or obesity and the detrimental health effects that result in decreased survival, as shown in this study.

## Data availability statement

The original contributions presented in the study are included in the article/Supplementary material, further inquiries can be directed to the corresponding author.

## Author contributions

CM obtained VetCompass data. CM, BM, and MS: study conception and design. BM organised the database. Analysis and interpretation of results was performed by BM and MS. Initial manuscript draft was written by BM, which was critically reviewed by MS to produce the final manuscript. All authors contributed to the article and approved the submitted version.
